# Perceptions of Wind Turbine Noise and Self-Reported Health in Suburban Residential Areas

**DOI:** 10.3389/fpsyg.2021.736231

**Published:** 2021-08-30

**Authors:** Fei Qu, Aki Tsuchiya

**Affiliations:** ^1^School of Architecture and Urban Planning, Shenzhen University, Shenzhen, China; ^2^Department of Economics, School of Health and Related Research, University of Sheffield, Sheffield, United Kingdom

**Keywords:** annoyance, health impact, focusing effect, wind turbine noise, questionnaire survey

## Abstract

Wind turbines play an important role in the worldwide mission of producing renewable energy. The development toward integrating large-scale wind turbines in the urban environment has raised concerns over the noise impacts on urban residents. While most of the existing studies on wind turbine noise (WTN) have focused on rural settings, this paper investigates the relationship between WTN, noise perception and self-reported health of people, and controlling for background characteristics of the residents in urbanized areas. Questionnaire surveys were carried out around three suburban wind farms in the UK with 359 respondents. A-weighted sound pressure levels of WTN were predicted using noise mapping, for the most exposed façade of each dwelling of the respondent. The dose-response relationship was found between WTN and annoyance, moderated by age and degree of education. WTN was associated with some aspects of self-reported health, including raised health concerns, having headaches, nausea, and ear discomfort, but was not related to sleep disturbance directly. Noise sensitivity, attitudes to wind energy, and visibility of the wind turbines were found to significantly influence self-reported health. By employing a second variant of the questionnaire with the research aim masked, this study also addressed the focusing effects induced by the questionnaire design. The significant differences in the reported adverse health between questionnaire variants implied focusing bias among the sample who knew the research purpose. This elicited a methodological finding that should be noted in future research.

## Introduction

Wind turbines (WTs) play an important role in producing renewable energy and mitigating greenhouse gas emissions. In recent years, there has been a development toward integrating large-scale WTs within the urban environment (Ishugah et al., 2014), which can reduce electricity loss and network costs due to its proximity to users (Archer and Jacobson, 2007; Hoppock and Patiño-Echeverri, 2010). However, noise pollution to the surrounding premises can be obstacle to wind energy exploitation. Noise emission from a WT is larger than the typical urban noise sources and consists of dominant components at low frequencies (below 200 Hz), which is attenuated less by buildings than mid- to high-frequency sound (Bolin et al., 2011).

The potential noise impacts of WTs have attracted substantial public, policy, and scientific attention. A limited number of cross-sectional studies have conducted questionnaire surveys to investigate the impact of wind turbine noise (WTN) on self-reported noise evaluations. Dose-response relationships between the exposure to WTN and annoyance have been found in five studies conducted in Sweden, the Netherlands, Poland, and Canada (Pedersen and Waye, 2004, 2007; Pedersen et al., 2009; Pawlaczyk-Łuszczyńska et al., 2014; Michaud et al., 2016). In addition, a dose-response relationship between self-reported sleep disturbance and noise exposure was found (Pedersen, 2011; Bakker et al., 2012; Pawlaczyk-Łuszczyńska et al., 2014). Other health-related effects such as psychological distress were found to be associated with WTN, with noise annoyance as a mediator (Pedersen, 2011; Shepherd et al., 2011). However, much of the existing research has focused on rural settings, with suburban respondents being a minority. Results from previous studies on the WTN evaluation in urbanized areas have been in a state of flux. One study found that living in an urbanized area (as opposed to a rural area) reduced the risk of annoyance with WTN (Pedersen and Waye, 2007), and another study found that living in a built-up area increased the probability of being annoyed (Pedersen et al., 2009), while another article of people living in noisy areas reported that noise exposure did not lead to noise annoyance among those who noticed the sound (Bakker et al., 2012). It has been inconclusive that urban residents were more vulnerable or adaptive to WTN than rural residents. One reason might be the existence of a complex set of socioeconomic parameters in urban areas that had not been fully controlled for in previous studies. Perceptions of noise could also be moderated by the visual aspects (Bangjun et al., 2003; Maffei et al., 2013; Aletta et al., 2016), vegetation (Van Renterghem, 2019), and the existing background noise (Qu and Kang, 2019). Given the increase in the WT size and the number of built-up areas, there is a need to investigate the perception and health impact of WTN in urbanized environments, controlling for respondent demographical and attitudinal factors.

Furthermore, previous surveys have asked respondents living near WTs to assess the impact of WTN directly (Pedersen and Waye, 2004, 2007; Pedersen et al., 2009; Pawlaczyk-Łuszczyńska et al., 2014). Therefore, it would have been obvious to the respondents that the purpose of the questionnaire was to investigate potential adverse health effects of WTs (Nissenbaum et al., 2012), and if so, such questionnaires may be susceptible to a focusing bias (Wilson et al., 2000; Ubel et al., 2011), where the questions lead the respondents to pay more attention than they usually do to the noise and thus answer differently. Therefore, there is a need for a questionnaire survey designed to avoid possible focusing bias.

This study aims to model the distribution of WTN in the suburban–urban residential areas and to investigate the relationships between the modeled exposure to WTN and noise perceptions, self-reported sleep disturbance, and health of the respondents. Noise annoyance of this study in the suburban–urban areas is compared to that in previous studies in rural areas. The work also explores if demographical and attitudinal factors affect reporting of noise impacts by the respondents. In addition, the study is designed to minimize the potential bias caused by focusing effects by using two variants of the questionnaire: one with and another without specific questions on WTs. These two variants of the questionnaire allowed an investigation on whether the knowledge of the motivation of the survey affects the reporting of health impacts by respondents.

## Materials and Methods

### Questionnaires

The study used a questionnaire survey of those living near WTs to investigate the relationship between the exposure to WTN, noise perceptions, and the self-reported health of the respondents. The questionnaire asked about the responses to WTN, sleep disturbance, the prevalence of health-related problems, and general health. It also measured the socioeconomic status of a respondent, architectural factors, attitudes to environmental issues, as well as visibility of the WT (full questionnaires are shown in Supplementary Material Sections 1, 2). In general, the questionnaire had three sections in the following order: (i) well-being and health, (ii) evaluation of the neighboring environment (including WTN), and (iii) sociodemography and dwelling. Most of the questions were drawn from the established national surveys of health and well-being such as the British Household Panel Survey (BHPS), with several modifications to fit this survey.

#### Questionnaire Variants

To minimize the potential focusing bias caused by the knowledge of the motivation of the survey, two variants of the questionnaire were used: “Questionnaire Variant 1” included questions on noise perceptions, personal attitudes, and health problems related to WTs, while “Questionnaire Variant 2,” allocated to a control group, which had no reference to WTs except in one question where WTs were referred to as one of several environmental nuisances. Other questions that did not refer to WTs were kept identical across the variants.

#### Outcome Variables

The main outcome variables included the perception of WTN, self-reported sleep disturbance, perceived health impact, the prevalence of specific health symptoms, and general health status. Perceptions of residents on WTN were assessed in a set of contingency questions adapted from a previous survey (Pedersen and Waye, 2004). Respondents were first asked to indicate whether they noticed any of the seven environmental nuisances including WTN, and if yes, they were asked to rate their degree of annoyance on a 5-point scale from “not at all” to “extremely.” In Variant 1, the annoyance with WTN was further examined in three questions: addressing annoyance overall, outdoors, and indoors. Sleep disturbance in this survey was measured without referring to noise and was kept identical in Variants 1 and 2. Unlike the previous studies that investigated the occurrence of disturbed sleep by noise using a single question (Pedersen and Waye, 2004, 2007; Bakker et al., 2012), the present study assessed the occurrence of various types of sleep disturbances such as difficulty in falling asleep, sleeping less deeply, and awakening. Further, the participants were asked to indicate whether they experienced the listed 10 physiological and psychological health problems during the past week, including headache, dizziness, ear discomfort, cardiovascular disease, tension and edginess, and lack of concentration. In Variant 1, the respondents were then allowed to indicate whether they felt WTN might be the cause. The response scale was configured as “yes,” “possibly,” “no,” and “I don't know.” In addition, all respondents were asked to self-assess their general health on a five-point scale from poor to excellent.

#### Moderating Variables

As for moderating variables, the survey included questions on sociodemographic, personal/attitudinal, and architectural factors. First, sociodemographical factors such as age, sex, longstanding illness, and household income that were found to influence noise annoyance and health in previous studies were assessed (Fields, 1993; Bluhm et al., 2004; Dolan et al., 2008; Frijters and Beatton, 2012). Second, personal/attitudinal questions addressing personal noise sensitivity, environmentally sustainable lifestyle, and attitude to the noise source were added in line with the previous studies (Weinstein, 1980; Guski, 1999; Job, 1999). Noise sensitivity was measured in one question with two items: (a) “I find it hard to relax in a place that's noisy” and (b) “I get used to most noises without much difficulty,” assessed on a 6-point scale from agree strongly to disagree strongly. The question was drawn from an established 21-item noise sensitivity questionnaire (Weinstein, 1978), shortened in this following questionnaire (Benfield et al., 2014). The attitude of respondents to WTs was also assessed using eight antonym adjectives to describe WTs, drawn from a previous study (Pedersen and Waye, 2004). A question identified the financial stakes of respondents in the wind farm. Furthermore, three questions were on architectural factors, such as the number of bedrooms in the dwelling, the housing type, and the orientation of the dwelling, which were found to have effects on resisting the WTN in previous studies (Qu and Kang, 2017). Furthermore, dwelling-related questions measured the visibility of the WT, length of residency, and ownership of the dwelling.

A detailed report on the questionnaire design can be found in Qu and Tsuchiya (2018).

### Study Sites and Sample

The target population of the survey was defined as the residents who lived within 2 km of modern WT(s) in suburban areas in the UK. The selection of each sample is explained below.

#### Study Area

**Figure 1:**
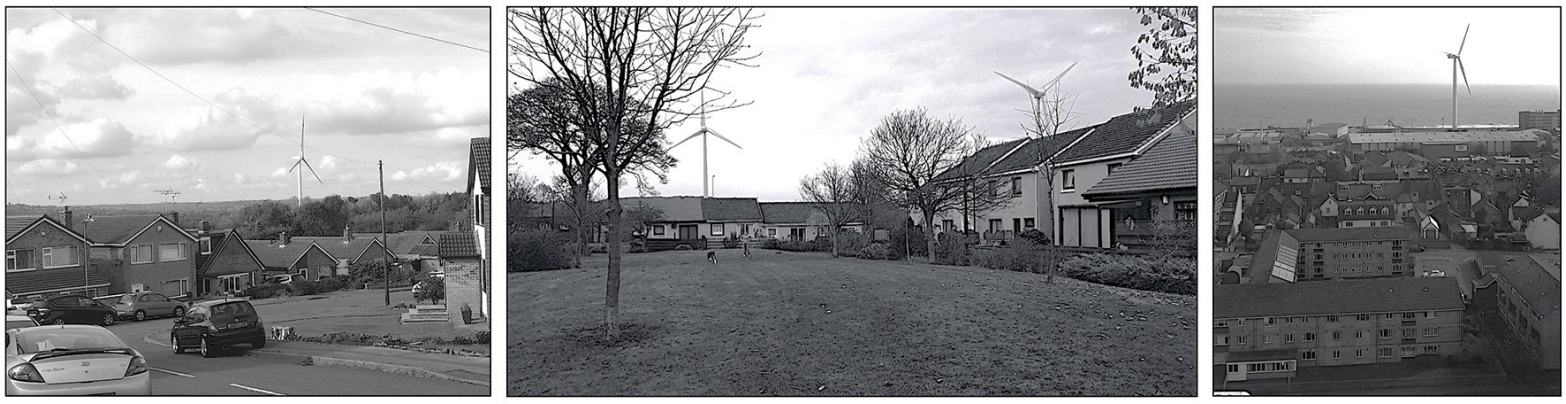
Photos of three study sites (left: Site A with wind turbine (WT) in the field; middle: Site B with WTs on the industrial site; right: Site C with WT at the seaside; photos were taken by the author).

Three typical suburban sites with modern WTs were selected, based on the UK wind energy online database (UKWED, 2014) and a map of each wind farm site on Google Earth. The photos of the three sites are shown in Figure 1. Site A is in the suburban area near Nottingham in East Midlands, site B is in the suburb of a Dundee city in Scotland, and site C is in the town of Lowestoft on the eastern coast of England. The WTs were large and modern, with tower heights between 80 and 85 m. The distances between the sampled residences and the closest WT were within 500–2,000 m. All sites could be classified as suburban with high population densities (2,000–4,000/km^2^). Site selection criteria and detailed site maps are shown in Supplementary Material Section 3.

#### Study Sample

To ensure that residents exposed to different levels of noise were represented in the sample, disproportionate stratified sampling was applied with modeled WTN levels as the strata. Preliminary noise modeling was carried out to predict the distributions of WTN across the residential areas of each site, considering different WT models and terrain conditions. The sample was sorted according to 5 dB noise intervals calculated from the noise maps (shown in Supplementary Material Section 3). The sample size in each stratum was calculated based on the power analysis (sample size calculation in Supplementary Material Section 4). Addresses were randomly selected from the edited version of the electoral register, by stratum, for each of the two questionnaire variants. All addresses in the highest noise-exposed group were included to reach the proportionate sample. Where there were several adults at the same address, one individual was selected at random. To save on the labor and cost of a survey, fewer samples were selected for the control group of Variant 2. As there were insufficient or unreachable addresses in some strata, a total of 2,971 individuals were sampled (2,238 for Variant 1 and 733 for Variant 2). Questionnaires were mailed or door-dropped to the sampled individuals.

### Noise Exposure Modeling Using the Noise Map Technique

In this article, WTN exposure was represented by the modeled maximum sound pressure level (SPL) at each dwelling of the respondents. To examine the spatial distribution of WTN levels in each study site, noise maps were calculated using the software package CadnaA (DataKustik GmbH, 2006). The map and topographical information of the study sites were obtained from the EDINA Ordnance Survey Digimaps in the UK (Ordnance Survey, 2013). A-weighted SPLs on the most exposed façade (maximum façade exposures) of target buildings were predicted. The calculation in the software was based on the ISO 9613-2 (ISO 9613-2, 1996) sound propagation standard. Noise emission from the WT was calculated under downwind conditions representing the worst case. In line with the IEC 61400-11 standard (IEC 61400-11, 2012), the WT was simulated as a point source at hub height. The sound power level and the spectrum of the point source were set based on that given by the manufacturer, where the sound powers are relatively high at low frequencies and attenuate with octave. The ground absorption was set to 0.5 in accordance with the Good Practice Guidelines in the UK (Cand et al., 2013). The temperature in software calculation was set to 10°C and the relative humidity was set to 70% for atmospheric absorption, which was consistent with common practices (Keith et al., 2016). The reflection order by buildings was set to 3, based on a previous study (Kang, 2006). The receiver was set at a 4 m height, with a 0.05 m façade-receiver distance, to take into account reflections at the exposed façade. After setting all the parameters, the building evaluation in the software generated the maximum SPL at each dwelling. The noise exposure for respondents was obtained based on their location on the noise maps. The calculations using the above method have been verified by field measurements. Results of validation showed that the software model provided an accurate estimate of the relative difference between locations around a building, especially at the middle-higher frequencies.

### Statistical Analysis

Statistical analyses were performed using the SPSS version 22 (Statistics, 2009). Descriptive statistics was provided for the characteristics of the participants, their attitudes to WT projects, their perceptions of WTN, and their self-reported health. Differences in the distribution of respondent characteristics and perceptions across four sound intervals and between two variants were examined using Pearson's chi-square (χ^2^) for categorical variables or ANOVA (*F*, in one-way ANOVA) for continuous variables. A nonparametric approach was also applied as an alternative to one-way ANOVA, using the Kruskal–Wallis test. Pearson's correlation coefficient (r) was used to test the bivariate correlation between noise exposure and subjective factors.

Binary logistic regression was applied to analyze the effects of noise exposure on annoyance with the noise, sleep disturbance, and perceived health impact. As very few respondents were highly annoyed in this study, annoyance measured on verbal scales was dichotomized with slightly annoyed to extremely annoyed classified as “annoyed.” The main explanatory variable, noise exposure, was represented by the A-weighted SPL, calculated for the most exposed façade of a dwelling from the noise map outlined above. Preliminary regression analyses were carried out to explore the influence of demographical, architectural, and attitudinal factors on noise perception and select the variables for the final regression models. Odds ratios (ORs) are reported for variables in each regression model with 95% CIs, with a *p*-value of below 0.05 considered to be statistically significant. The Nagelkerke pseudo-*R*^2^ was applied as a measure of the explained variance. The Hosmer–Lemesow goodness-of-fit [*p* (H-L)] was presented for each logistic regression model, with a *p*-value of >0.05 indicating no statistically significant difference between the modeled and the observed data.

## Results

### Descriptive Statistics

#### Respondents

The numbers of respondents of the two questionnaire variants were 262 and 97, respectively, with a total of 359. The overall response rate was 12.0%. The response rates of Variants 1 and 2 were similar, of 11.7 and 13.2%, respectively. Based on a chi-squared test of goodness-of-fit, the distribution of the respondents according to 5-dB(A) noise intervals was not statistically different across questionnaire variants, χ^2^(7) = 3.34, *p* = 0.343.

Across the two variants, the mean age in the study population was 56 (*SD* = 17.7), and 49% were male. Most of the respondents were employed (43%) or retired (41%). Overall, 49% of the respondents lived in detached or semidetached houses, and 68% of the respondents privately owned their accommodation. No respondents in this study had a financial stake or were employees of the local wind farm. The characteristics of respondents were similar across the two variants.

Respondents were not evenly distributed across the four noise groups. In general, respondents exposed to high levels of noise pollution were more vulnerable than those living in lower sound residences, shown to be older (*F*_(3, 352)_ =9.87, *p* < 0.001), retired (χ^2^(7) = 13.23, *p* = 0.004), widowed (χ^2^(7) = 15.38, *p* = 0.002), living in a rented flat (χ^2^(7) = 30.61, *p* = 0.002), and had lower household income (*r* = −0.19, *p* = 0.050). This might be due to the planning of wind farm areas where residences in proximity to the turbines were more often rented by people from a low social class, while lower sound residences were detached or semidetached with more bedrooms and were more likely to be owned by mid-to-high class people. These demographical factors were controlled for in the relationship between WTN exposure and potential health effects.

#### Perception of WTN

Overall, 16% of the respondents (*n* = 59) noticed the WTN and 11% of the respondents (*n* = 39) were being annoyed by it when asked alongside a set of environmental nuisances. Of those who noticed WTN, 41% were not annoyed by the noise.

It was found that 80% of the annoyed respondents were living within 850 m, and 90% were living within 900 m from the WT. When the WT was over 900 m away from the residence, the percentage of respondents who noticed and were annoyed decreased to 8.1 and 2.7%, respectively.

When respondents in Variant 1 were asked further about their annoyance with WTN in a separate question, 12% (*n* = 32) indicated that they were annoyed by the noise overall, 16% (*n* = 45) were annoyed outdoors, and 9% (*n* = 25) were annoyed indoors. In terms of sound characters, more than half of the respondents of Variant 1 (55%) described the WT as noiseless/quiet. Swishing (29%) and whooshing (20%) were the most common sound characteristics chosen, which are verbal descriptors of low-frequency components of the sound from WTs.

Across the two variants, there were no differences in the proportions of respondents who noticed noise from WTs (χ^2^(3) = 0.09, *p* = 0.763) or those who indicated to be annoyed (χ^2^(3) = 0.04, *p* = 0.837).

#### Attitudinal and Visual Factors (Variant 1 Only)

Participants in Variant 1 were asked for their judgments on WTs using 14 adjectives (Question No. 15 in the questionnaire of Variant 1 shown in Supplementary Material 1). The adjectives that most of the respondents agreed with were “environmentally friendly,” (71%) “efficient,” (41%) “necessary,” (38%) and “harmless” (37%). “Ugly” was the most frequently selected among the negative adjectives (23%).

Factoring analysis was employed to extract the oblique factors underlying the 14 inter-related adjectives. Five factors were identified; three of them were significantly related to noise annoyance. One factor was a positive attitude to the utility of WTs (*r* = −0.14, *p* = 0.023), described as environmentally friendly, efficient, harmless, and natural. Another factor was a negative attitude to the necessity of exploiting wind energy (*r* = 0.22, *p* < 0.001), which was expressed as unnecessary and threatening. The last factor was a negative attitude to environmental impacts (*r* = 0.34, *p* < 0.001), including not environmentally friendly, dangerous, ugly, and unnatural.

Respondents to Variant 1 were asked to indicate the visibility of WT(s) from their residence; 31% (*n* = 80) responded that they could not see any from their home; 31% (*n* = 80) could only see WT(s) from a window; 12% (*n* = 30) could only see it/them from the garden or front yard; and 25% (*n* = 66) could see WT(s) from both a window and the garden/yard. Respondents who could not see any WT from home were associated with a lower proportion of annoyance than those who could see the turbine (3.8 vs. 14.8%, χ^2^(3) = 6.65, *p* = 0.010), while those who could see the turbine from both a window and the garden significantly increased the proportion of annoyance (from 6.6 to 24.2%, χ^2^(3) = 15.55, *p* < 0.001).

#### Self-Reported Health

Respondents in both Variants 1 and 2 indicated sleep disturbances without referring to noise. Overall, 13.4% of the respondents did not have their sleep disturbed. The most often chosen problems were “sleeping less deeply” (33.1%) and “lie awake for a while” (32.6%). The proportion of disturbed sleep was not associated with noise exposure from the WTs.

Levels of general health were self-reported based on an established question using 5-scales from excellent to poor. A majority of the respondents reported their health as good (*M* = 2.92, *SD* = 0.98). No statistically significant differences were found related to the general health between the questionnaire variants or noise exposure groups.

The perceived health impacts of respondents in Variant 1 showed different distributions across four noise groups. The proportion of respondents who indicated that noise from the WT(s) had no effect on their health varied from 93.8 to 92.1% at low SPLs but decreased to 77.3% at SPLs >40 dBA; such a difference between sound categories was statistically significant (χ^2^(7) = 10.50, *p* = 0.015).

**Figure 2:**
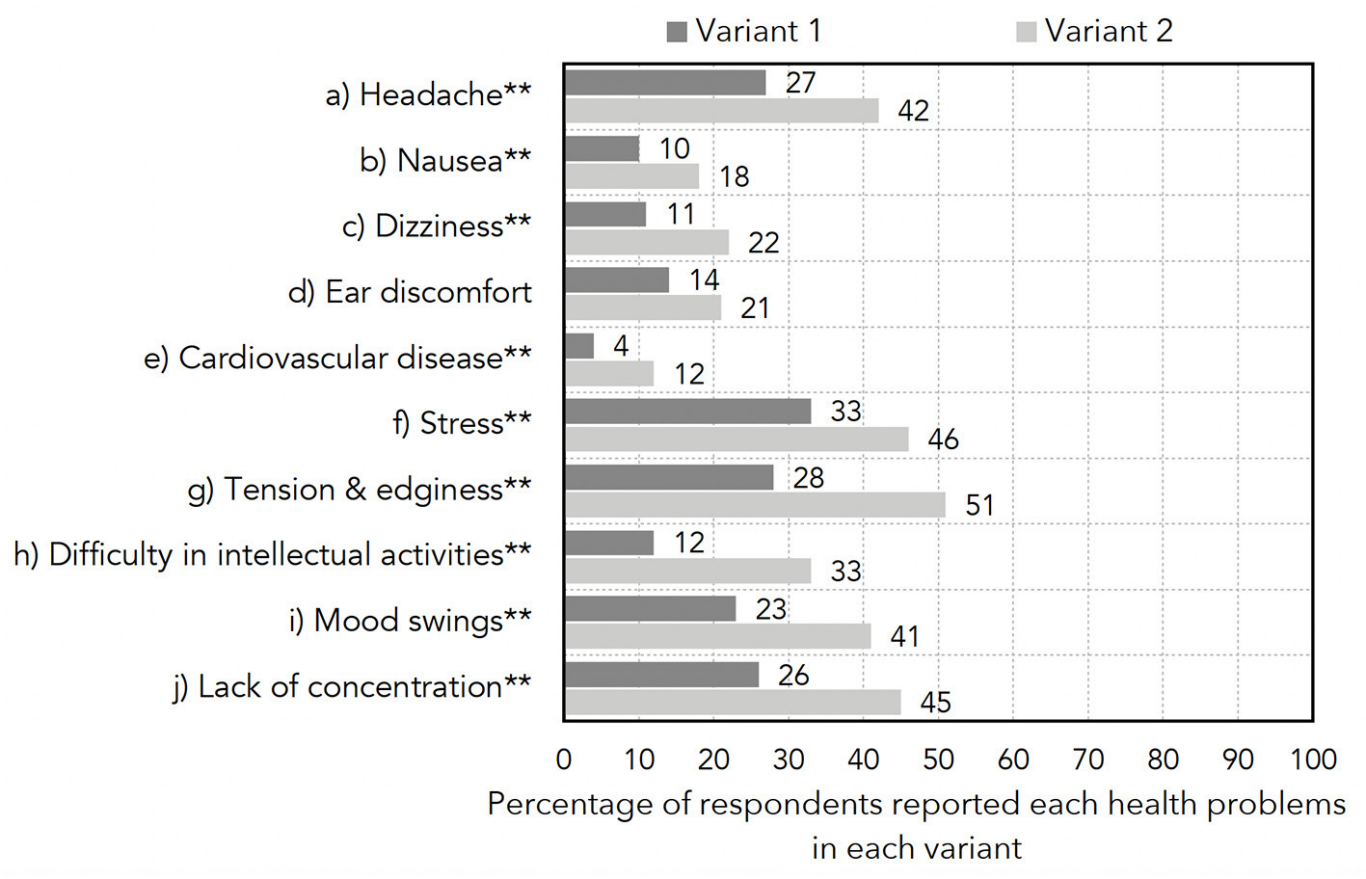
Clustered bar chart showing the percentage of respondents reporting health problems in Variants 1 and 2 (**significant differences across variants with *p* < 0.05 for chi-squared tests).

Respondents in both Variants 1 and 2 indicated whether they experienced any of the listed health symptoms during the past week, such as headache, nausea, dizziness, and stress. The percentage of respondents in each variant who experienced each health symptom is shown in Figure 2. The prevalence of the reported health symptoms was higher in Variant 2, which might be due to a significantly higher proportion of respondents having a long-standing illness or disability in Variant 2. Another reason might be that some respondents in Variant 1, knowing that the motivation of the survey was to link their reported health symptoms to WTN, under-reported their health problems unless they thought that WTN might be the cause.

### Dose-Response Relationships Between Noise Exposure and Perception

Table 1 shows the percentage of respondents noticed and annoyed by WTN in each group of 5 dB sound intervals. The proportion of “noticed” respondents increased from 5 (*n* = 5) at a sound interval below 30 dBA to 47% (*n* = 25) at a sound interval above 40 dBA. The proportion of annoyed respondents also increased with sound interval, from 3% (*n* = 3) in the lowest to 30% (*n* = 16) in the highest. Chi-squared tests show that the differences between sound intervals were statistically significant.

**Table 1:** Annoyance with wind turbine noise (WTN) related to sound exposures is shown as the percentage within each sound interval with 95% CI.

	**Overall**	**Maximum sound pressure levels at dwelling [dB(A)]**				**Chi-squared test**
		**<30**	**30–35**	**35–40**	**>40**	
**Variants 1+2**	**Percentage (95% CI)**	**Percentage within each sound interval (95% CI)**				
Noticed (among other nuisances)	16 (13–20)	5 (1–11)	12 (6–19)	20 (12–29)	47 (33–61)	χ^2^ = 45.056; *p* = 0.000
Annoyed overall (among other nuisances)	11 (8–15)	3 (0–6)	8 (3–14)	13 (7–21)	30 (17–43)	χ^2^ = 24.598, *p* = 0.000

### Effects of WTN on the Perception and Health Controlling for Personal Factors

#### Annoyance With WTN

Binary logistic regression was used to examine the association between noise exposure, personal factors, and annoyance. In preliminary regression analyses, the modeled maximum SPL at the dwelling was kept in each regression model as the main explanatory variable; personal factors that were hypothesized to have an effect were then added to the regression model one by one. Eighteen preliminary regression models were run to select the independent variables to be used in the main multivariate model (results of these preliminary regression analyses are shown in Supplementary Table 1). It is worth noting that annoyance with WTN was not associated with sex or income and was not different statistically among vulnerable respondents who had a long-standing illness, who were being retired or on maternity leave, or whether they owned their dwellings. Self-reported noise sensitivity, which significantly influenced noise annoyance in previous studies, was not found to have a significant impact on noise perceptions in this study. Architectural factors, including the number of bedrooms, housing type, and orientation, were not associated with annoyance.

The multivariate regression models predicted annoyance using the moderating variables that had a significant influence in the bivariate models, shown in Table 2. The OR of being annoyed by WTN increased with each dB increase in SPLs at the most exposure façade. Age was positively associated with annoyance at a diminishing rate. Having higher educational qualifications than A-level or O-level decreased the probability of being annoyed. Visibility of the WT from both window and garden increased the odds of being annoyed than not visible from home (*p* < 0.10). Holding a negative attitude to the environmental impact of WTs was positively associated with annoyance.

**Table 2:** Association among annoyed by WTN, sound pressure levels (SPLs), and covariates.

**Model**	**Variables**	***p*** **-value**	**Odds Ratio (OR)**	**95% CI for OR**
	***Annoyed by WTN** [n = 356, R^2^ = 0.264, p_(*H*−*L*)_ = 0.308]*			
*1*	SPL	0.000	**1.18**	(1.08–1.28)
*(Variants 1+2)*	Age	0.011	**1.24**	(1.05–1.47)
	Age squared	0.006	**0.81**	(0.69–0.94)
	*Highest qualification (ref: A-level or O-level)*			
	- No qualification	0.153	0.49	(0.18–1.31)
	- Higher education below degree	0.077	**0.31**	(0.08–1.14)
	- Degree level	0.047	**0.25**	(0.06–0.98)
	- Other (professional certificate)	0.602	1.51	(0.32–7.22)
	Variant 2	0.799	0.89	(0.38–2.11)
	***Annoyed by WTN** [n = 254, R^2^ = 0.339, p_(*H*−*L*)_ = 0.331]*			
*2*	SPL	0.050	**1.12**	(1.00–1.26)
*(Variant 1)*	Age	0.025	**1.24**	(1.03–1.48)
	Age squared	0.016	**0.80**	(0.67–0.96)
	*Highest qualification (ref: A-level or O-level)*			
	- No qualification	0.167	0.40	(0.11–1.48)
	- Higher education below degree	0.039	**0.22**	(0.05–0.93)
	- Degree level	0.073	**0.25**	(0.06–1.14)
	- Other (professional certificate)	0.634	1.69	(0.20–14.41)
	***Variables only in Variant 1 below:***			
	*Visibility of the WT (ref: cannot see any from home)*			
	- See WT from the window	0.249	2.43	(0.54–10.98)
	- See WT from the garden	0.851	0.82	(0.10–6.80)
	- See WT from both the window and the garden	0.062	**4.81**	(0.93–24.95)
	Negative attitude to the environmental impact of WT (no/yes)	0.001	**4.84**	(1.84–12.73)

*Statistically significant associations with p < 0.10 in boldface*.

#### Sleep Disturbance

Sleep was not related to WTN but annoyance with the noise. The annoyance of a respondent was positively associated with sleeping less deeply both for the whole data and for the main sample of Variant 1 (Table 3). Fixing the degree of annoyance overall, higher age, and having a long-standing illness increased the odds of sleeping less deeply. Being female and sensitive to noise did not make a significant difference. Of the models on the sample of Variant 1, the visibility of the WT from both a window and garden significantly increased the odds of a less deep sleep by 2.78 times than those who only saw it from a window.

**Table 3:** Association among sleep, WTN annoyance, and covariates.

**Model**	**Variables**	***p*** **-value**	**Odds Ratio (OR)**	**95% CI**
1	***Sleep less deeply (no/yes)** [n = 335, R^2^ = 0.110, p_(*H*−*L*)_ = 0.827]*			
(Variants 1+2)	SPL	0.317	0.98	(0.94–1.02)
	Annoyance overall (scale 1–5)	0.024	**1.54**	(1.06–2.25)
	Age	0.057	**1.02**	(1.01–1.04)
	Female	0.599	0.88	(0.54–1.42)
	Longstanding illness (no/yes)	0.035	**1.69**	(1.02–2.78)
	Sensitivity to noise (scale 1–6)	0.369	1.08	(0.92–1.27)
	Variant 2	0.148	0.66	(0.38–1.16)
2	***Sleep less deeply (no/yes)** [n = 242, R^2^ = 0.209, p_(*H*−*L*)_ = 0.949]*			
(Variant 1)	SPL	0.234	0.97	(0.91–1.02)
	Annoyance overall (scale 1–5)	0.021	**1.83**	(1.11–3.03)
	Age	0.058	**1.03**	(1.01–1.05)
	Female	0.973	0.99	(0.54–1.80)
	Longstanding illness (no/yes)	0.013	**1.86**	(1.00–3.44)
	Sensitivity to noise (scale 1–6)	0.930	0.99	(0.81–1.22)
	Negative attitude to the environmental impact of WT (no/yes)	0.781	1.10	(0.58–2.09)
	*Visibility of the WT (ref: see WT from window)*			
	- Cannot see WT	0.198	1.67	(0.77–3.62)
	- See WT from the garden	0.755	0.85	(0.29–2.44)
	- See WT from both the window and the garden	0.011	**2.78**	(1.20–6.42)

*Statistically significant associations with p < 0.10 in boldface*.

#### General Health and Perceived Health Impact

Wind turbine SPL was not significantly associated with the self-reported general health levels but might affect the perceived health impact of a respondent among the sample of Variant 1. To explore the influence of subjective factors on the perceived health impact, binary multiple logistic regression was used (Table 4). Two models were created: one containing WT SPL at the dwelling as the prime variable and the other containing both SPL and annoyance. As shown in Table 4, WTN exposure increased the level of health concerns. When adding annoyance of WTN into the model, the effect of SPL was still significant, though not at the 0.05 level. Respondents who were annoyed by WTN were much less likely to report no health concerns than those not annoyed. Age and having positive attitudes to the utility of WTs increased the odds of reporting no health impact. Holding negative attitudes to the necessity or environmental impact of the WT did not significantly increase health concerns. Being female significantly decreased the odds of reporting no health impacts.

**Table 4:** Association among no health concerns, sound pressure levels (SPLs), and covariates.

**Model**	**Variables**	***p*** **-value**	**Odds Ratio**	**95% CI**
	***Perceived no health impact** [n = 255, R^2^ = 0.203, p_(*H*−*L*)_ = 0.672]*			
1	SPL (maximum)	0.012	**0.89**	(0.81–0.97)
(Variant 1)	Age	0.034	**1.03**	(1.00–1.06)
	Female	0.038	**0.34**	(0.12–0.94)
	Positive attitude to the utility of WT (no/yes)	0.018	**4.36**	(1.29–14.69)
	Negative attitude to the necessity of WT (no/yes)	0.169	0.38	(0.10–1.51)
	Negative attitude to the environmental impact of WT (no/yes)	0.951	0.97	(0.32–2.89)
	***Perceived no health impact** [n = 255, R^2^ = 0.252, p_(*H*−*L*)_ = 0.833]*			
2	SPL (maximum)	0.053	**0.91**	(0.83–1.00)
(Variant 1)	Age	0.053	**1.03**	(1.00–1.06)
	Female	0.022	**0.28**	(0.10–0.84)
	Positive attitude to the utility of WT (no/yes)	0.016	**4.91**	(1.35–17.93)
	Negative attitude to the necessity of WT (no/yes)	0.244	0.42	(0.10–1.82)
	Negative attitude to the environmental impact of WT (no/yes)	0.695	1.26	(0.40–4.01)
	***Annoyed by WTN overall (no/yes)***	0.008	**0.22**	(0.07–0.67)

*Statistically significant associations with p < 0.10 in boldface*.

#### Self-Reported Health Symptoms

Noise exposure from WTs was positively related to the prevalence of headache, nausea, and ear discomfort, but only within the respondents of Variant 2. Table 5 shows the binary logistic regression models with a significant association between SPL and a health symptom among the sample of Variant 2. As the socioeconomic status of a respondent was related to WTN exposure, the regression controlled for age, sex, household income, and self-reported noise sensitivity. The results suggested that age slightly decreased the odds of headache and nausea but increased the probability of having ear discomfort. Other things being equal, the female was 2.7 times more likely to report headaches than the male, while the self-evaluated noise sensitivity level was positively associated with reporting health symptoms. All models had relatively high levels of *R*^2^, indicating that more than 38% of the variance in headache and 51% of the variance in nausea could be explained by SPLs and the personal variables in the regression model.

**Table 5:** Association among health problems, WTN (SPLs), and covariates in Variant 2.

**Model**	**Variables**	***p*** **-value**	**Odds Ratio (OR)**	**95% CI for OR**
1	***Headache** [n = 97, R^2^ = 0.385, p_(*H*−*L*)_ = 0.791]*			
	SPL	0.071	**1.100**	(0.99–1.22
	Age	0.087	**0.970**	(0.93–1.01)
	Female	0.077	**2.752**	(0.89–8.45)
	Household income	0.833	0.941	(0.54–1.65)
	Sensitivity to noise (scale 1–6)	0.001	**2.126**	(1.35–3.35)
2	***Nausea** [n = 97, R^2^ = 0.519, p_(*H*−*L*)_ = 0.012]*			
	SPL	0.038	**1.250**	(1.01–1.54)
	Age	0.004	**0.907**	(0.85–0.97)
	Female	0.140	3.969	(0.63–24.81
	Household income	0.193	0.530	(0.20–1.38)
	Sensitivity to noise (scale 1–6)	0.056	**2.712**	(0.98–7.54)
3	***Ear discomfort** [n = 97, R^2^ = 0.280, p_(*H*−*L*)_ = 0.509]*			
	SPL	0.059	**1.118**	(0.99–1.25)
	Age	0.030	**1.068**	(1.01–1.13)
	Female	0.597	1.413	(0.39–5.09
	Household income	0.830	1.083	(0.52–2.25)
	Sensitivity to noise (scale 1–6)	0.021	**1.883**	(1.10–3.21)

*Statistically significant associations with p < 0.10 in boldface*.

## Discussion

### Comparison With Previous Studies in Rural Areas

This study has found that the risk of annoyance with WTN increased with the modeled noise levels at a dwelling, which confirms the dose-response relationship found in previous studies (Pedersen and Waye, 2004, 2007; Pedersen et al., 2009; Pawlaczyk-Łuszczyńska et al., 2014; Michaud et al., 2016). Comparing the results of this study to those in rural areas, WTN in urbanized areas of this study is less noticeable than those in rural areas. Higher levels of WTN could annoy more rural residents than those of suburban inhabitants. The findings correspond well with Bakker et al. (2012) that further analyzed the data of Pedersen et al. (2009), covering both rural and built-up areas and indicated that the risk of being disturbed and distressed by WTN is pronounced in quiet areas compared to noisy areas.

The reason for the above differences between the current study and previous ones could be explained from both acoustical and contextual aspects. From the acoustical aspect, the study sites of the current study were more urbanized than those of the previous studies. In urbanized areas, higher levels of road traffic and neighborhood noise can have a masking effect on WTN (Qu and Kang, 2019). It is also possible that WTN is less prominent than other nuisances and stressors in a suburban area; the most frequently reported being the barking of dogs, racing cars, and motorcycles in this study. To explain the difference from the contextual aspects, respondents in suburban areas of this study seemed to be optimistic about new clean energy devices, which is supported by the free comments left at the end of the questionnaire, where many of them gave positive comments on wind energy and referred to various sustainable lifestyles such as fitting solar panels. The beliefs of people about the importance of the source of the noise could decrease annoyance, as stated in the literature (Fields, 1993).

### Effects of Moderating Factors

The degree of noise annoyance can considerably vary between individuals of different characteristics, as identified in the literature (Weinstein, 1980; Fields, 1993; Guski, 1999). In this study, it is important to note that the background characteristics of respondents were significantly different across noise categories, where respondents in the higher exposure group were also lower in the sociodemographic status. This increased the probability of multicollinearity. Efforts had been made to assess the effects of WTN controlling for a series of demographic, attitudinal, architectural, and visual factors. The results suggest that the characteristics of residents such as age, gender, education, and noise sensitivity significantly affect the degree of individual noise perception or self-reported health, most of which were not reported as significant in previous WTN studies.

Negative attitudes to the environmental impact of WTs, described as not environmentally friendly, dangerous, and ugly, were positively associated with the risk of annoyance. It is consistent in previous studies that the negative attitudes to WTs especially to their visual impacts increase the possibility of annoyance (Pedersen and Waye, 2004, 2007; Pawlaczyk-Łuszczyńska et al., 2014).

Having at least one WT visible from the dwelling has been found to increase noise annoyance in a previous study (Pedersen and Waye, 2007). Among the respondents of this study, visibility of the WT did increase annoyance compared to not being able to see any but was not statistically significant. This may be interpreted along with the previous finding that the visual impact was more pronounced in rural areas when compared to that in the more densely populated areas (Pedersen and Larsman, 2008). However, a significantly higher annoyance was found among those suburban respondents who can see the WT from both a window and the garden, where the WT might be perceived as more obvious and contrasting with the landscape, which may lead to more annoyance, as stated by previous investigations (Pedersen and Larsman, 2008; Maffei et al., 2013).

### Effects of WTN on Self-Reported Health

It has been found that noise levels were not associated with sleep but the degree of noise annoyance significantly increased the possibility of sleeping less deeply. The results agree well with the previous findings that WTN does not directly influence sleep, but annoyance acts as a mediator (Bakker et al., 2012). However, it should be noted that a reverse causality from sleep to annoyance might exist. The absence of a significant association between noise levels and sleep in this study might also be because urban respondents were more adaptive to noise. According to the findings of a meta-analysis study, a dose-response relationship between self-reported sleep disturbance and A-weighted noise exposure was not found in more densely populated suburban areas with various sound sources (Pedersen, 2011).

It was found that both the WTN level and annoyance increased perceived health impacts within the studied sample, controlling for sociodemographic variables. Being male, age growth and a positive attitude to wind energy project significantly moderated the health concerns, which has not been addressed in previous studies.

Self-reported general health was not found to be related to the WTN level nor the annoyance with the noise but was related to socioeconomic factors such as household income and the presence of illness within the studied sample. One possible reason is that the noise effect on subjective health and well-being might take more time to appear than the effects on annoyance. It is also possible that the level of general health might be related to other contextual factors that were not included, such as urbanization (Hudson, 2006), trust (Helliwell, 2006), and individual adaptation (Luhmann et al., 2012).

The association between SPL and the self-reported headache, nausea, and ear discomfort is in line with the literature that environmental noise with low-frequency components such as aircraft noise was more likely to increase the risk of headache and irritability (Stansfeld et al., 2000). The effects of WTN on dizziness and ear discomfort have been pointed out in several reports based on the complaints of local residents (Harry, 2007; Thorne and Leader, 2012), but have not been found in the previous field studies.

### Effect of Questionnaire Variants

An important finding of the study lies in the difference between the two groups. Adverse health problems were more frequent in Variant 2 for whom the research purpose was masked. No significant associations were found between the noise level and the prevalence of health problems among respondents in the main group of Variant 1. A reversed focusing effect might exist in some participants of Variant 1, showing under-reported health problems. The reason could be related to the effect of the questionnaire design that has informed the participants in Variant 1 that their health data would be analyzed in relation to WTN. This might have led to fewer health problems being reported by the respondents of Variant 1, as 89% of them had indicated that WTN did not influence health. Another possible reason might be that the respondents of Variant 1 living in the low exposure zones over-reported their health symptoms, as the survey asked them to attribute the cause of any health symptom to WTN, which made them focus on the adverse impact of WTN on health and introduced bias. This behavior has been reported in a previous study on aircraft noise, that the wording of specific questions aimed at eliciting symptoms had a marked effect on the answers (Barker and Tarnopolsky, 1978). However, the differences in adverse health impacts between Variants 1 and 2 implied that results in Variant 1 with symptoms attributed to noise might represent focusing effects based on the knowledge of a respondent rather than on real noise effects.

In previous studies, the substantial questions on attitudinal and visual aspects of the WT in the same questionnaire implied the research topic to respondents. In this situation, the questions get the respondents to focus on WTN, and respondents might choose the item they thought was most relevant to the study. The usefulness of the two variants is a methodological finding, which is important to be noted.

### Practical Implications of the Finding

The findings of this article can be utilized to guide the planning authorities to define suitable areas for the placement of WTs within the existing suburban contexts. From the perspective of noise management, urbanized landscapes are considered more suitable for one or two stand-alone modern WTs than rural landscapes with natural values. The separation distance for one or two WTs in urbanized areas is suggested to be at least 900 m. As in this study, 80% of the annoyed respondents were living within 850 m, and 90% were living within 900 m from the WT. From an architectural perspective, it is suggested to develop apartment buildings to attract younger and highly educated residents. Garden areas and bedroom windows are best to be at the quiet side of the building, opposite the WT. This can reduce the visibility of the turbine from both a window and the garden, which was found to be more annoying in this study. In addition, as negative attitudes were found to significantly influence subjective evaluations of WTN, public participation in an early stage of the planning might be useful, such as consultations and site visits that could change the adverse impression of a resident and build public trust.

### Limitations and Future Works

The study had several limitations, which could be worthwhile for future work. One limitation related to the noise mapping was that the study only considered the WTN exposure in the worst case, such as in downwind conditions and with an 8 m/s wind velocity for the near-maximum noise output. Using one calculated SPL value to represent a certain situation might introduce inaccuracy, as the SPL could vary during the day and night due to atmospheric conditions. Future work can estimate an average level of SPL using the yearly statistics of wind speed, which might be more appropriate to predict the long-term noise annoyance.

Another limitation of this survey was, as with the previous cross-sectional studies, that establishing causality was difficult. It is worth noting that for this study, it was difficult to isolate the effect of the noise itself due to the high positive correlation between the increased noise and the decreased socioeconomic status. Although many socioeconomic characteristics were controlled for, the inter collinearity between factors might change the impact coefficient of noise. Future works could conduct longitudinal studies over some time to investigate the long-term noise effects on subjective well-being. Future studies could investigate the effects of more moderating factors including the possibility of accessing to quiet side and the visibility of green areas in an urban context.

## Conclusion

Wind turbine noise exposure was positively associated with the self-reported noticeability and annoyance due to the noise. However, a higher level of noise seemed to generate less annoyance in urbanized areas of this study than that in rural areas, and the effects of personal factors could not be ignored. Annoyance due to WTN was found to be higher among older people and lower among those having higher education qualifications. Negative attitudes to the environmental impact of wind projects, judging them as dangerous, unnatural, and ugly, were positively associated with annoyance. Being able to see WT(s) from both a window and the garden/yard significantly increased the probability of being annoyed than those who could not see any from home.

This article found that WTN was associated with variations in some aspects of self-reported health, including raising health concerns, having headaches, nausea, and ear discomfort. It confirmed the findings of previous studies that sleep disturbance was not associated with noise levels directly but was related to noise annoyance and was moderated by age and long-standing illness.

This study established a method of employing a second variant of the questionnaire with the research aim masked to investigate the self-reported health symptoms and to reduce focusing bias. The main sample (Variant 1), who knew the research purpose, reported fewer health problems than the control group (Variant 2). A possible reason was that the questionnaire made these respondents focus on WTN and consider it as a source of ill health, which might induce the respondents to report symptoms based on their knowledge or assumptions of impacts and introduced focusing bias with over- or under-reported symptoms. It is suggested that future research could minimize the focusing bias by involving a control group with the research purposefully masked to differentiate the statistically modeled noise impact from the focusing impact of a respondent.

## Data Availability Statement

The raw data supporting the conclusions of this article will be made available by the authors, without undue reservation.

## Ethics Statement

The studies involving human participants were reviewed and approved by School of Architecture at the University of Sheffield. The patients/participants provided their written informed consent to participate in this study.

## Author Contributions

FQ carried out the noise mapping, resident survey, data analysis, and writing of the manuscript. AT participated in the supervision of the questionnaire design and statistical analyses, and reviewed and corrected the manuscript. Both authors approved the final version of the manuscript.

## Conflict of Interest

The authors declare that the research was conducted in the absence of any commercial or financial relationships that could be construed as a potential conflict of interest.

## Publisher's Note

All claims expressed in this article are solely those of the authors and do not necessarily represent those of their affiliated organizations, or those of the publisher, the editors and the reviewers. Any product that may be evaluated in this article, or claim that may be made by its manufacturer, is not guaranteed or endorsed by the publisher.
