# Supplementary material for: Perceptions of Wind Turbine Noise and Self-Reported Health in Suburban Residential Areas
Source: Front Psychol. 2021 Aug 30;12:736231. doi: 10.3389/fpsyg.2021.736231 (PMC8435591; doi:10.3389/fpsyg.2021.736231)
Supplement: Supplementary file 1 [file Presentation_1.PPTX]

## Slide 1
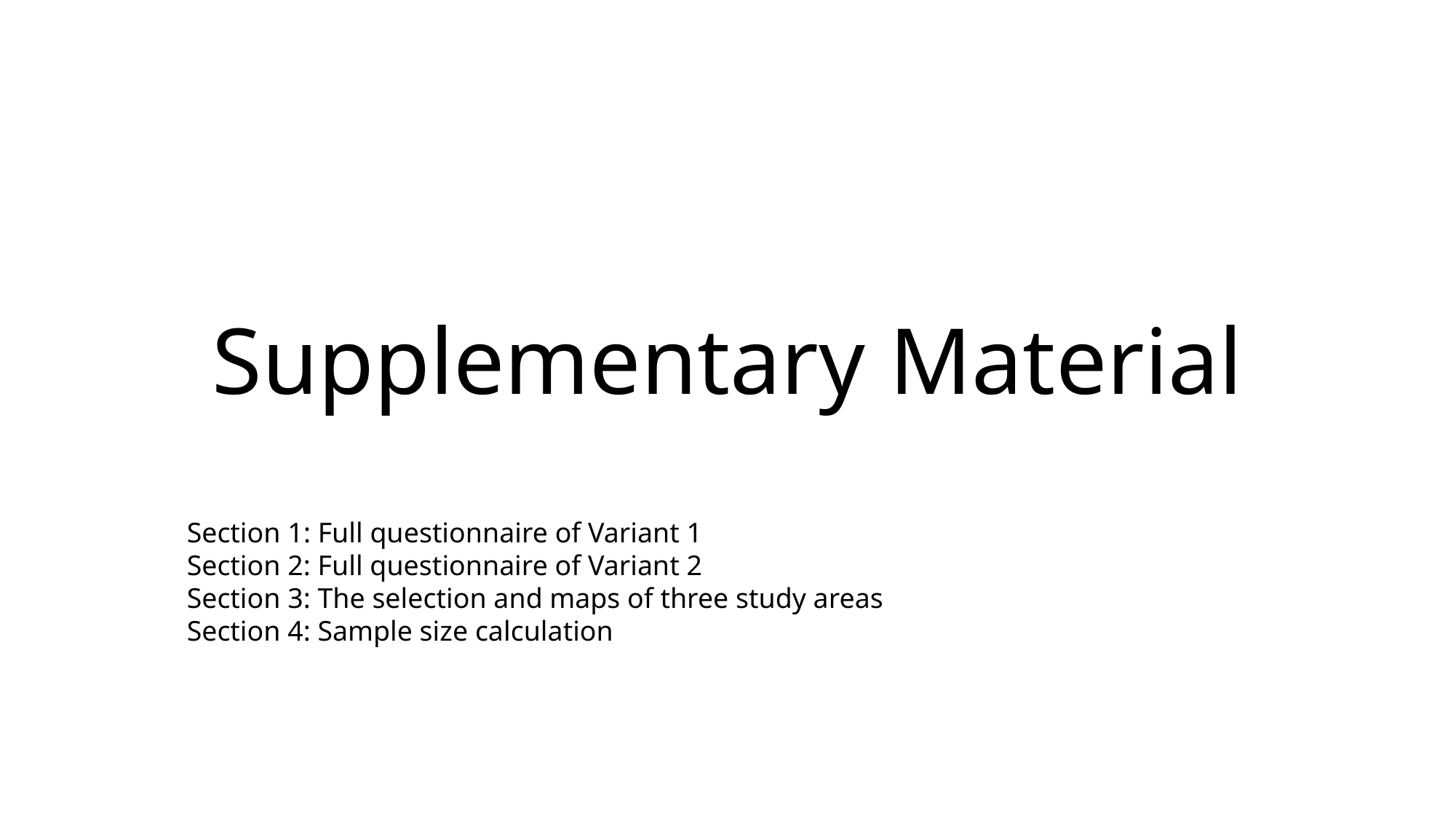

# Supplementary Material
Section 1: Full questionnaire of Variant 1
Section 2: Full questionnaire of Variant 2
Section 3: The selection and maps of three study areas
Section 4: Sample size calculation

## Slide 2
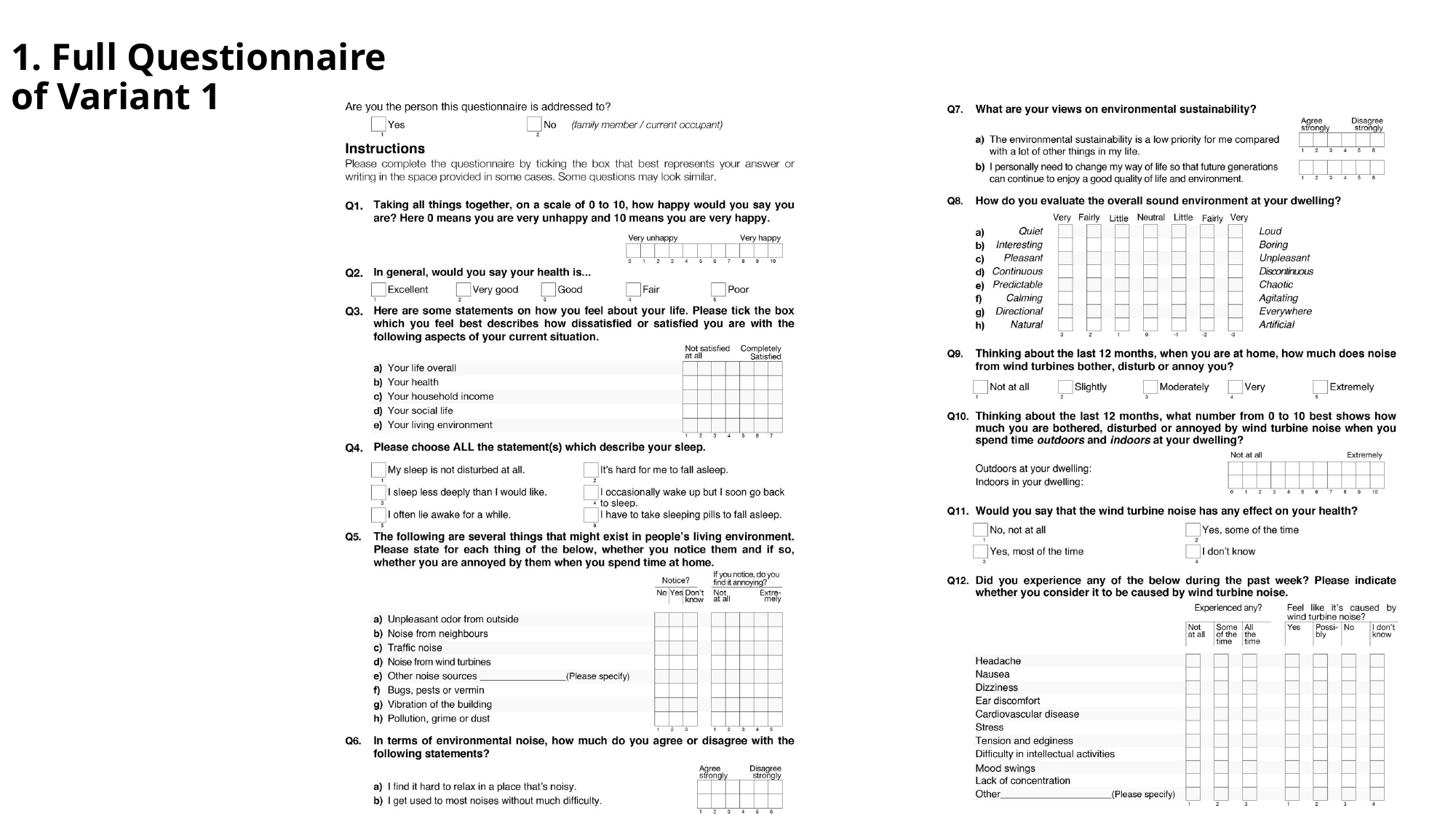

# 1. Full Questionnaire of Variant 1

## Slide 3
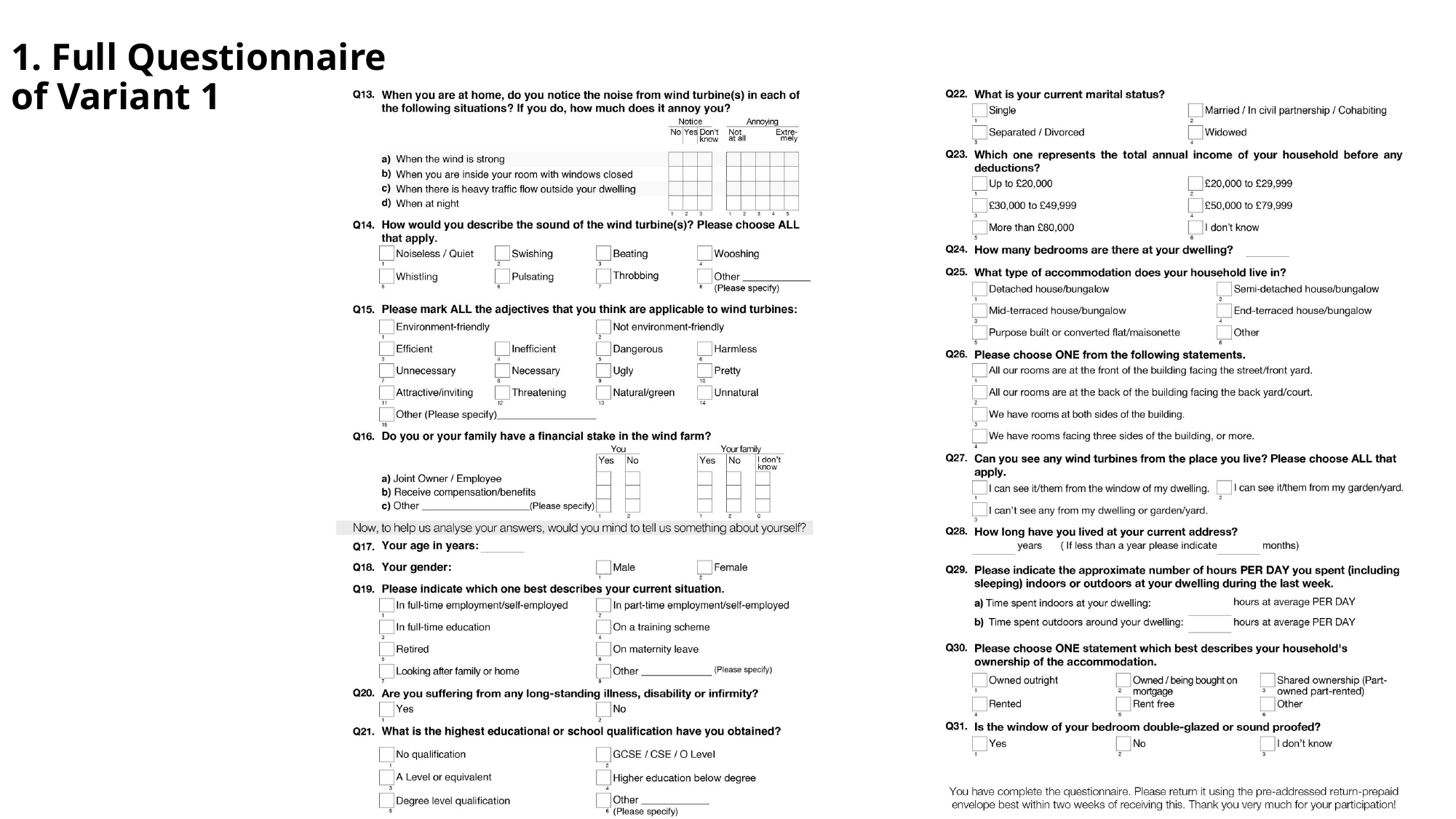

1. Full Questionnaire
of Variant 1

## Slide 4
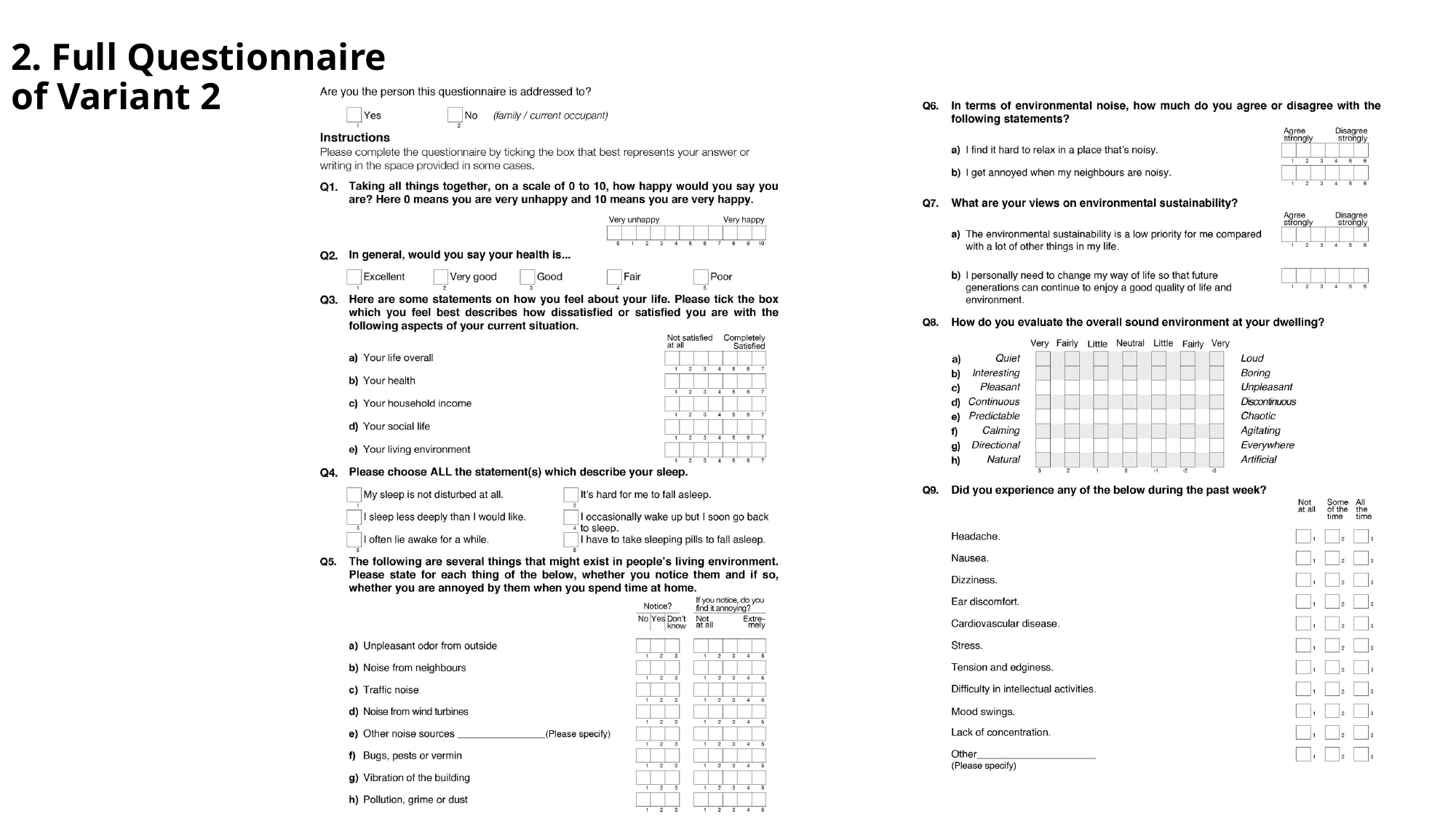

2. Full Questionnaire
of Variant 2

## Slide 5
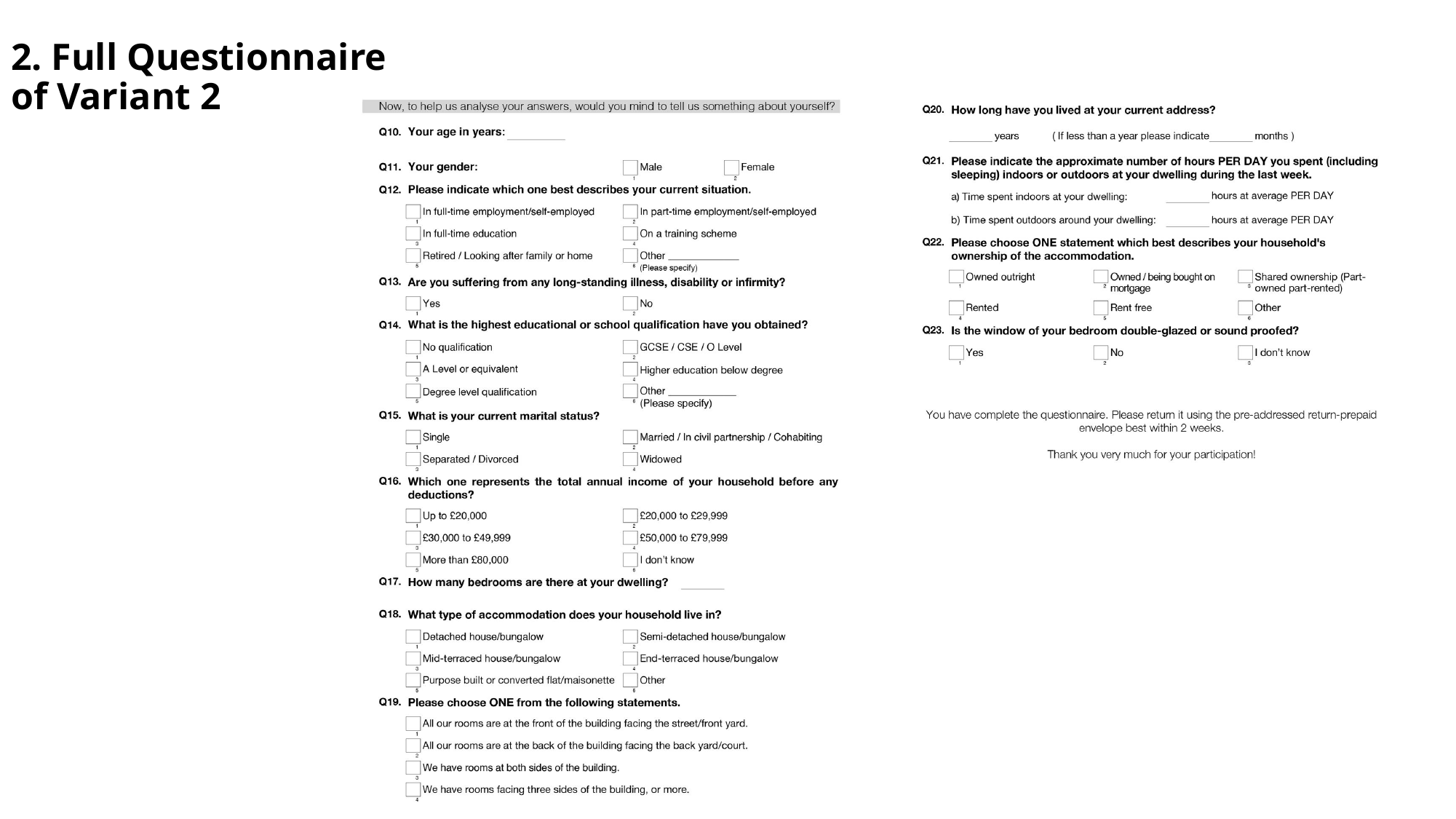

2. Full Questionnaire
of Variant 2

## Slide 6
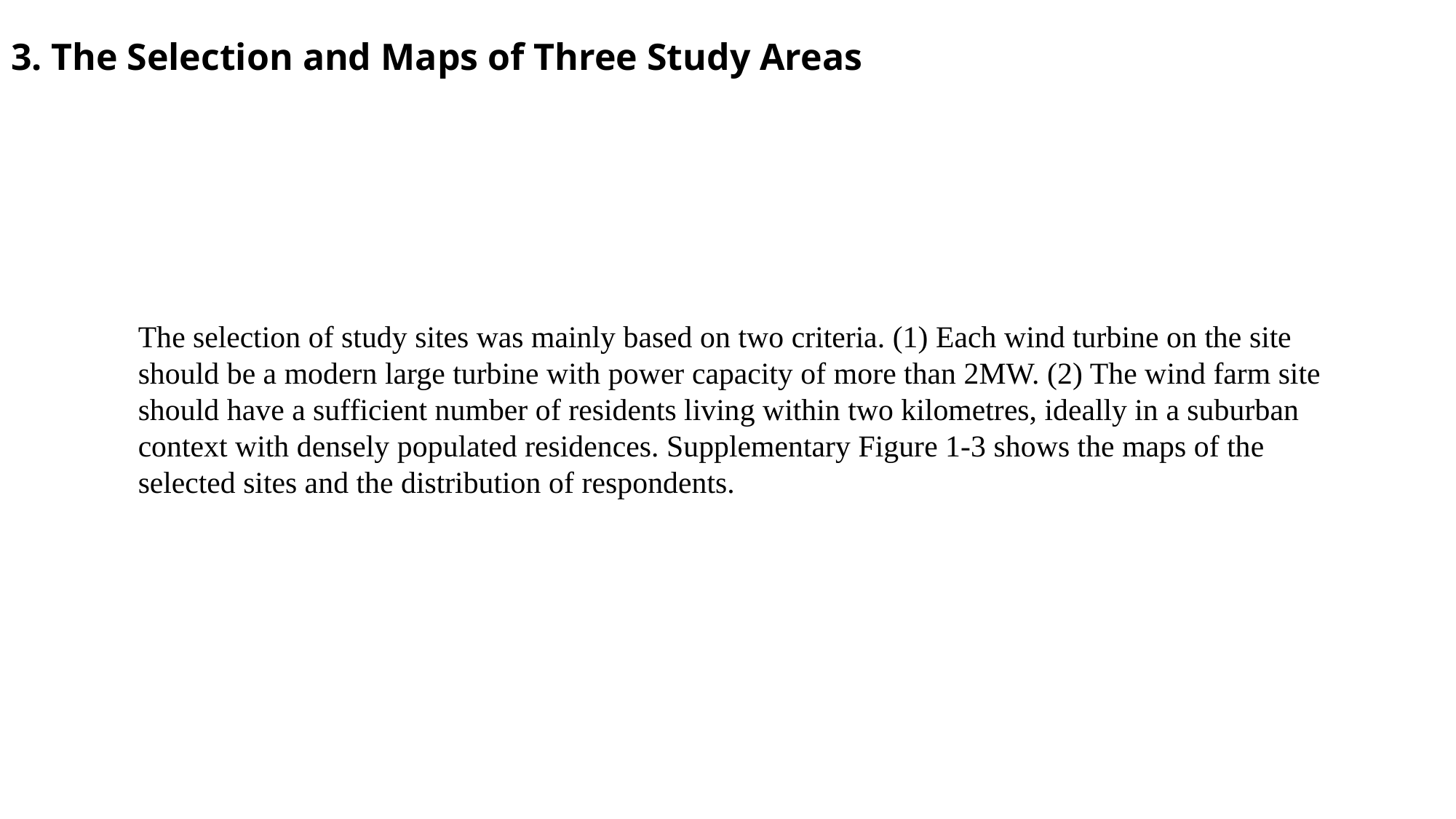

3. The Selection and Maps of Three Study Areas
The selection of study sites was mainly based on two criteria. (1) Each wind turbine on the site should be a modern large turbine with power capacity of more than 2MW. (2) The wind farm site should have a sufficient number of residents living within two kilometres, ideally in a suburban context with densely populated residences. Supplementary Figure 1-3 shows the maps of the selected sites and the distribution of respondents.

## Slide 7
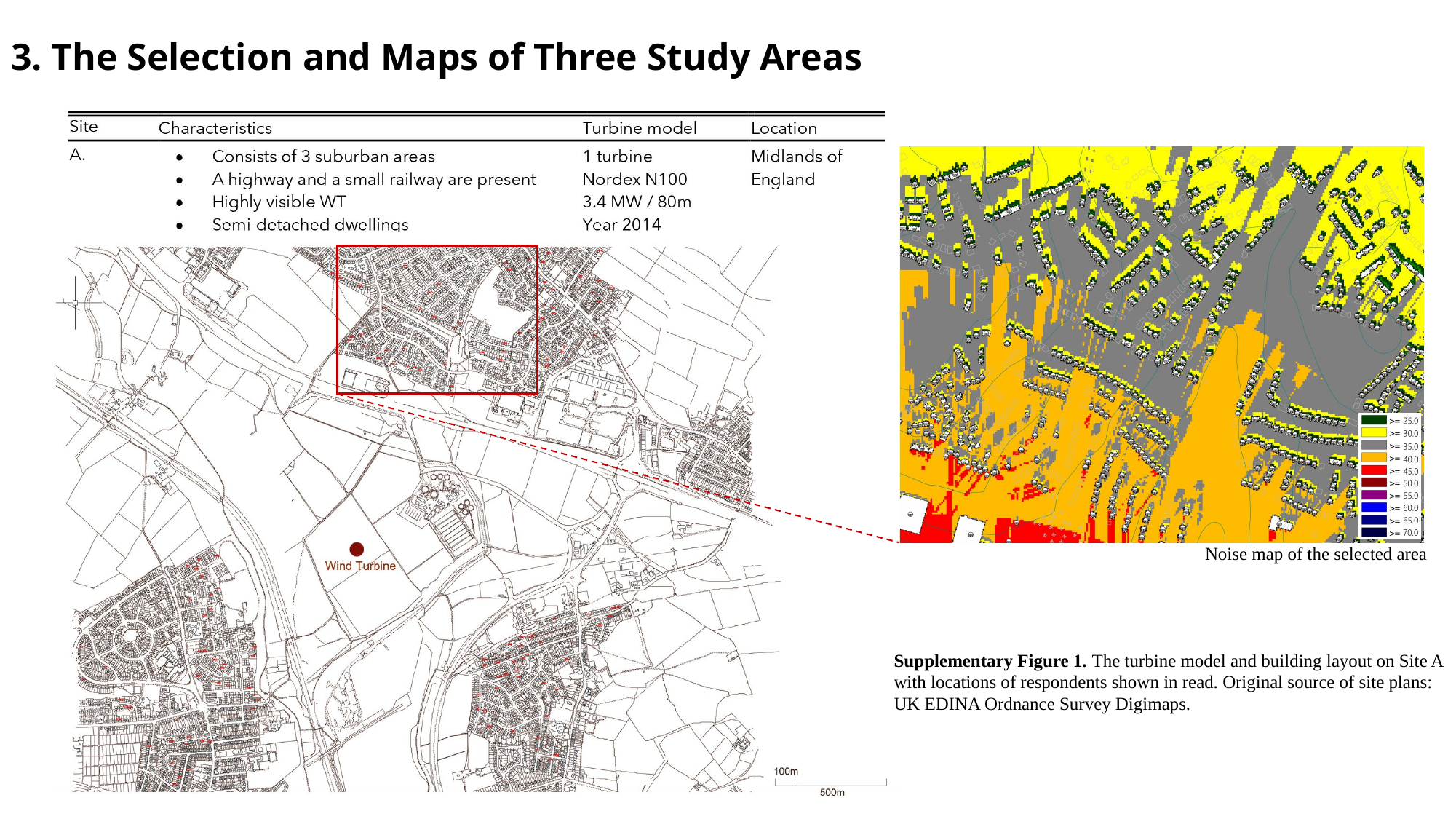

3. The Selection and Maps of Three Study Areas
Noise map of the selected area
Supplementary Figure 1. The turbine model and building layout on Site A with locations of respondents shown in read. Original source of site plans: UK EDINA Ordnance Survey Digimaps.

## Slide 8
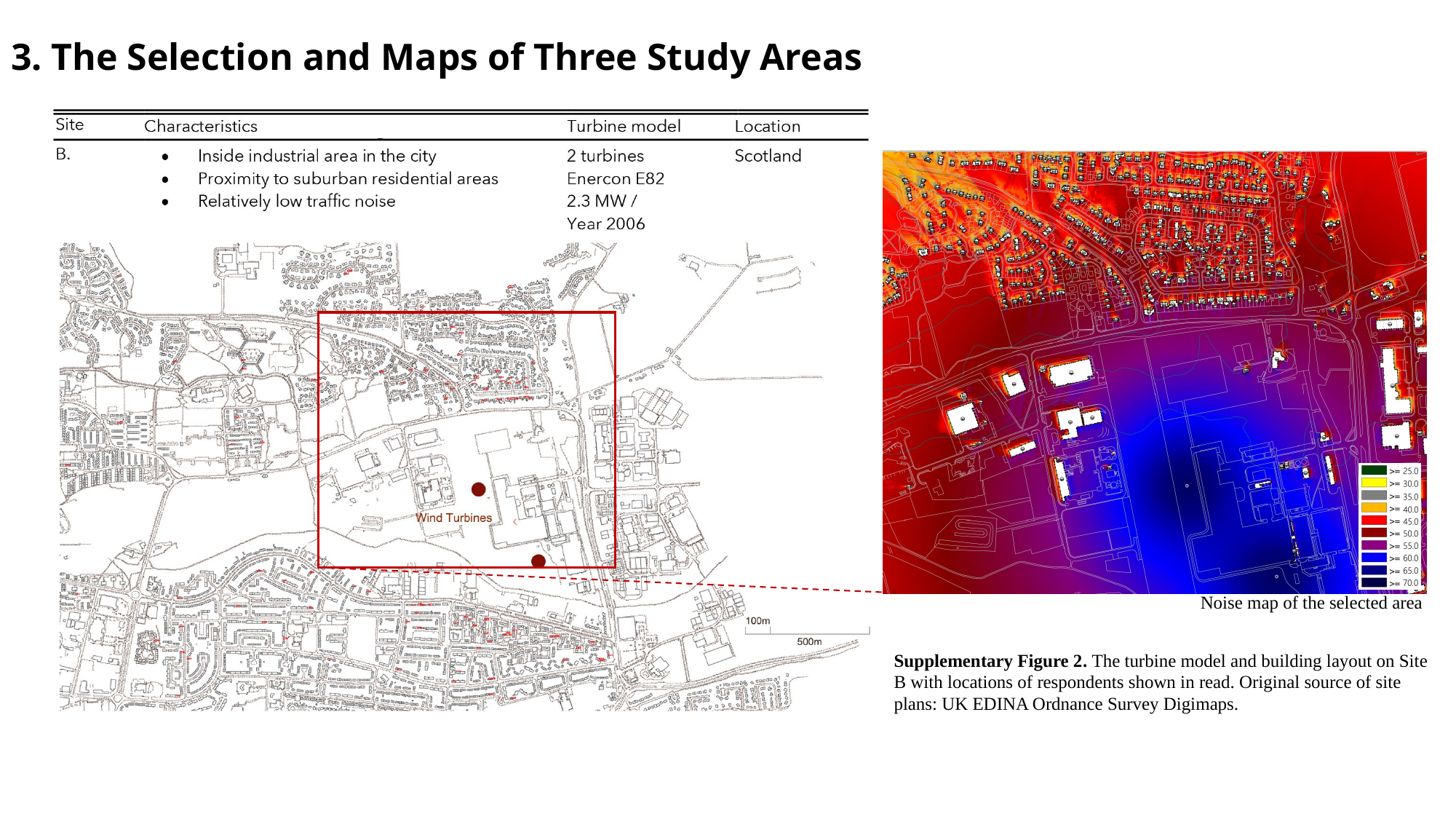

3. The Selection and Maps of Three Study Areas
Noise map of the selected area
Supplementary Figure 2. The turbine model and building layout on Site B with locations of respondents shown in read. Original source of site plans: UK EDINA Ordnance Survey Digimaps.

## Slide 9
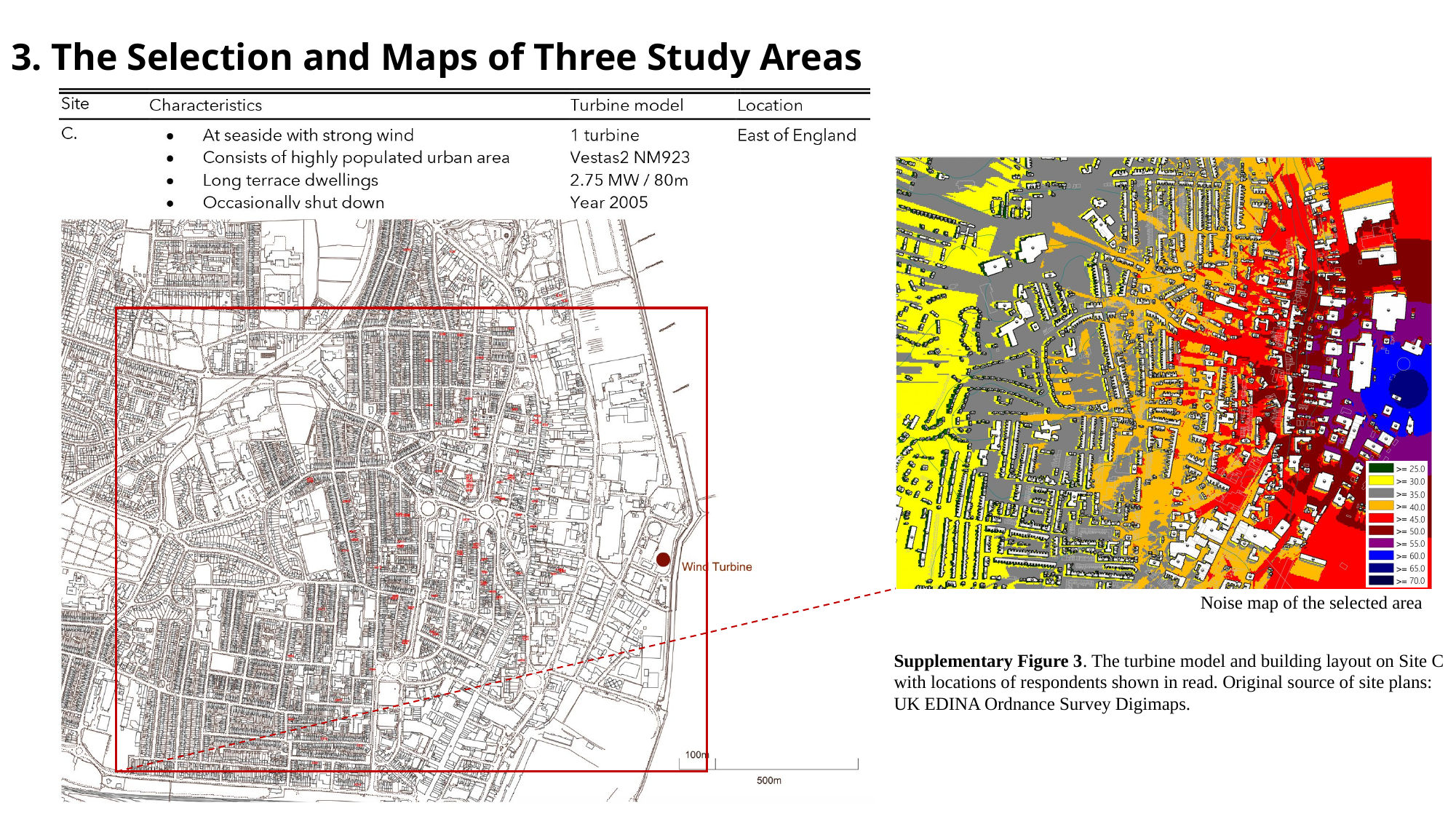

3. The Selection and Maps of Three Study Areas
Noise map of the selected area
Supplementary Figure 3. The turbine model and building layout on Site C with locations of respondents shown in read. Original source of site plans: UK EDINA Ordnance Survey Digimaps.

## Slide 10
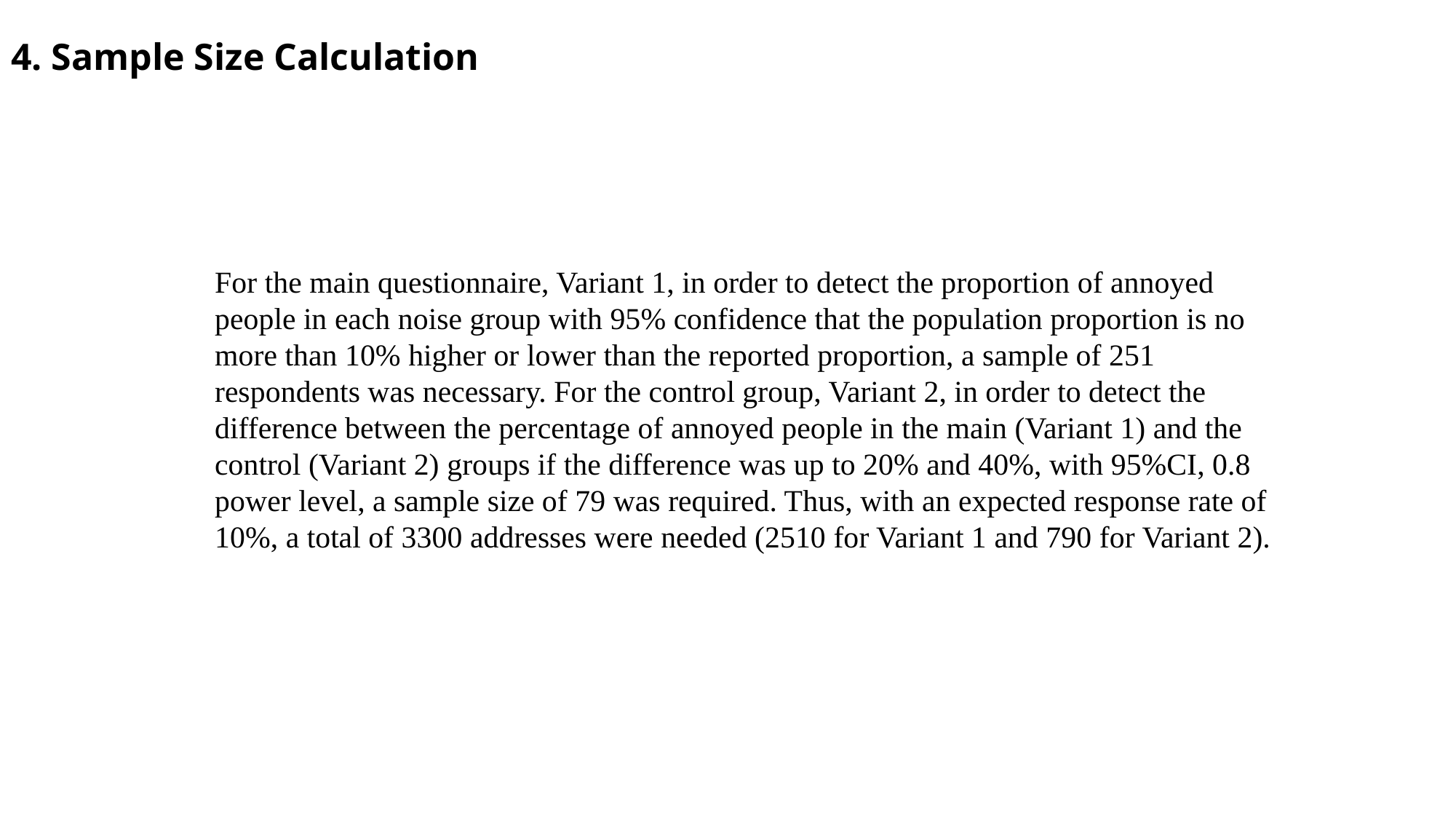

4. Sample Size Calculation
For the main questionnaire, Variant 1, in order to detect the proportion of annoyed people in each noise group with 95% confidence that the population proportion is no more than 10% higher or lower than the reported proportion, a sample of 251 respondents was necessary. For the control group, Variant 2, in order to detect the difference between the percentage of annoyed people in the main (Variant 1) and the control (Variant 2) groups if the difference was up to 20% and 40%, with 95%CI, 0.8 power level, a sample size of 79 was required. Thus, with an expected response rate of 10%, a total of 3300 addresses were needed (2510 for Variant 1 and 790 for Variant 2).
