# Supplementary material for: Perceptions of Wind Turbine Noise and Self-Reported Health in Suburban Residential Areas
Source: Front Psychol. 2021 Aug 30;12:736231. doi: 10.3389/fpsyg.2021.736231 (PMC8435591; doi:10.3389/fpsyg.2021.736231)
Supplement: Supplementary file 2 [file Data_Sheet_1.docx]

Supplementary Material

# Preliminary regression analyses on annoyance

Twenty-four regression models were created to explore the effect of moderating factors, as shown in Supplementary Table 1.The first model shows that the odds of being annoyed by wind turbine noise increased significantly with SPL. Subsequent models show that when demographic, attitudinal, and architectural factors were added one at a time as independent variables, the influence of the SPL remained statistically significant. Age and educational qualifications were significantly associated with annoyance (model 4, 6). Annoyance with wind turbine noise was not associated with sex or income, and was not different statistically among susceptible respondents who had long-standing illness, being retired or on maternity leave. Noise sensitivity and ownership of the dwelling was not associated with annoyance. Of the five factors measuring attitudes to wind farms in Variant 1, only the negative attitudes to environmental impact was significantly associated with annoyance (model 17).

Supplementary Table 1. Association between annoyance with wind turbine noise, sound pressure levels (SPLs), and covariates, expressed as odds ratio (OR) with 95% confidence intervals (CIs)

| Model | | SPL (dBA) | | Covariates of interest | | |
| --- | --- | --- | --- | --- | --- | --- |
| No. | R^2^ | OR | (95%CI) |  | OR | (95%CI) |
| 1 | 0.134 | **1.17** | **(1.09-1.25)** |  |  |  |
|  |  |  |  | ***Variant and site factors:*** |  |  |
| 2 | 0.137 | **1.17** | **(1.09-1.25)** | Questionnaire variant (Variant 2) | 1.11 | (0.50-2.44) |
| 3 | 0.146 | **1.15** | **(1.06-1.24)** | *Site (ref: Site C)* |  |  |
|  |  |  |  | - Site A | 1.21 | (0.42-3.47) |
|  |  |  |  | - Site B | 1.91 | (0.78-4.69) |
|  |  |  |  | ***Socioeconomic factors:*** |  |  |
| 4 | 0.213 | **1.20** | **(1.11-1.29)** | Age | **1.27** | **(1.08-1.29)** |
|  |  |  |  | Age squared | **0.79** | **(0.68-0.92)** |
| 5 | 0.137 | **1.17** | **(1.09-1.25)** | Sex (female) | 0.96 | (0.48-1.92) |
| 6 | 0.185 | **1.18** | **(1.10-1.26)** | *Highest qualification (ref: A-level or O-level)* |  |  |
|  |  |  |  | - No qualification | **0.26** | **(0.15-0.88)** |
|  |  |  |  | - A-level | 0.27 | (0.07-1.02) |
|  |  |  |  | - Higher education below degree | 0.41 | (0.12-1.40) |
|  |  |  |  | - Degree level | **0.24** | **(0.06-0.90)** |
|  |  |  |  | - Other professional/certification | 1.05 | (0.25-4.46) |
| 7 | 0.139 | **1.17** | **(1.09-1.27)** | Household income (low to high) | 0.98 | (0.66-1.44) |
|  |  |  |  | ***Vulnerable groups:*** |  |  |
| 8 | 0.135 | **1.17** | **(1.09-1.25)** | Sensitivity to noise (1-6) | 1.05 | (0.83-1.34) |
| 9 | 0.157 | **1.19** | **(1.11-1.27)** | Having long-standing illness | 0.75 | (0.36-1.56) |
| 10 | 0.157 | **1.19** | **(1.11-1.27)** | Retired | 0.82 | (0.44-1.53) |
| 11 | 0.138 | **1.17** | **(1.09-1.26)** | On maternity leave | 1.37 | (0.28-6.77) |
| 12 | 0.137 | **1.17** | **(1.09-1.26)** | Ownership of the dwelling (owned v.s. rent) | 1.12 | (0.54-2.33) |
|  |  |  |  | ***Attitude to & Visibility of the WT (only in Variant1):*** |  |  |
| 13 | 0.122 | **1.14** | **(1.06-1.23)** | Factor 1 (Positive to the utility) | 0.47 | (0.19-1.13) |
| 14 | 0.119 | **1.14** | **(1.06-1.23)** | Factor 2 (Positive to the appearance) | 0.37 | (0.08-1.65) |
| 15 | 0.126 | **1.14** | **(1.06-1.23)** | Factor 3 (Negative to the necessity) | 2.73 | (0.96-7.82) |
| 16 | 0.103 | **1.14** | **(1.06-1.23)** | Factor 4 (Negative to the efficiency) | 1.03 | (0.35-2.99) |
| 17 | 0.167 | **1.14** | **(1.06-1.23)** | Factor 5 (Negative to the environmental impact) | **3.44** | **(1.52-7.77)** |
| 18 | 0.156 | **1.10** | **(1.01-1.20)** | *Visibility of the WT (ref: can’t see any) (only in Variant 1)* |  |  |
|  |  |  |  | - See WT from window | 2.08 | (0.52-8.37) |
|  |  |  |  | - See WT from garden | 1.13 | (0.17-7.46) |
|  |  |  |  | - See WT from both window & garden | **4.40** | **(1.11-17.38)** |
| *Statistically significant correlations in boldface.* | | | | | | |
